# Commentary: On the possible role of stimulation duration for after-effects of transcranial alternating current stimulation

**DOI:** 10.3389/fnsys.2015.00148

**Published:** 2015-10-29

**Authors:** Kohitij Kar

**Affiliations:** Center for Molecular and Behavioral Neuroscience, Rutgers University - NewarkNewark, NJ, USA

**Keywords:** transcranial alternating current stimulation, entrainment, synaptic plasticity, EEG, alpha rhythm

In their recent article, Strüber et al. (2015) demonstrate that 1s application of transcranial alternating current stimulation (tACS) do not lead to any significant changes in the phase and amplitude of the electroencephalogram (EEG) signal. Therefore, they concluded that it is too short to induce synaptic plasticity. This is a very important observation that sheds light on possible underlying mechanisms of tACS. Although the results clearly show the absence of certain specific tACS-induced electrophysiological after-effects when applied only for 1s, some additional considerations need to be made in order to fully interpret these null results as well as probe the mechanism of tACS at shorter timescales.

An important question is whether at these smaller time scales, the lack of prolonged entrainment during the post stimulation session necessarily reflect a lack of tACS efficacy. Alternatively, these results might also suggest that for 1s stimulation paradigms, we are simply looking at the wrong electrophysiological measure. At these smaller time scales, changes in measures such as spike rate adaptation (Fernandez et al., 2011; Kar and Krekelberg, 2014), spike time precision (Reato et al., 2010), neurovascular coupling (Zheng et al., 2011; Kar and Wright, 2014) are likely to be more relevant for behavioral aftereffects. The EEG signal typically comprises of synchronized oscillations across the superficial cortex (for review, see Buzsáki et al., 2012). Hence it might be more sensitive to changes in entrainment whereas much less sensitive to these subtle effects (which might also still be behaviorally relevant). However, some of these changes might indeed be a result of changes in short-term synaptic plasticity (which is a very broad term). It must however be noted that the aforementioned mechanisms could also be a direct result of network entrainment during tACS but lack entrainment related features in the EEG measured at the scalp in the post tACS session. One way to test this hypothesis further, would be to use the method introduced by Helfrich et al. (2014) to remove the tACS-induced artifacts for the 1s tACS period, and estimate changes in EEG during stimulation. This would be very informative to test whether tACS applied at the individual alpha frequency (IAF) for 1s could entrain the underlying cortex at all. Then we would be able to say whether the lack of effect is despite similar entrainment during tACS.

The effects of tACS on the underlying cortex often depends on the presence of an experimental task that actively recruits the underlying brain area or otherwise, produces a specific brain state. For instance, Kar and Krekelberg (2014) demonstrated, that tACS induced changes in human motion discrimination performance is only present when tACS was paired with the visual motion stimulus. Ten Hertz tACS applied for 4s reduced the after-effects of motion adaptation and the effects scaled with how much adaptation there was to begin with. Similarly, Feurra et al. (2013) also demonstrated the state dependent effects of tACS on the motor cortex. Given this brain state dependency of the tACS induced effects, it remains unclear whether the lack of tACS-induced aftereffect reported in the study could be due to an absence of an appropriate brain state in the stimulated area. In this regard, it might be interesting to probe the brain areas while doing a relevant task.

Choosing the stimulation intensity is also a key consideration during tACS studies (Groppa et al., 2010). However, it is crucial to consider the confounding aspects of tACS-induced phosphenes (Kar and Krekelberg, 2012) and tactile sensations (Feurra et al., 2011). I argue that lower stimulation amplitudes might not necessarily control for phosphenes and these low amplitudes might fail to induce the desired cortical effects (entrainment). First, the accuracy of the threshold values are highly depended on the sensitivity of the adaptive method used to estimate them. In addition, we can never rule out the effects of subthreshold retinal stimulation during tACS. Hence, it is important to consider other strategies to design control experiments to rule out general effects of tACS (phosphenes, tactile sensations, reduced/increased arousal etc.). Some recent studies have used brain laterization to test their hypotheses (Kar and Krekelberg, 2014). But, given the large current spread during tACS and asymmetries between the two hemispheres of the human brain, this is not always feasible. Therefore, control strategies remain a challenging issue for tACS experiments.

The Strüber et al. (2015) study provides a lead into the hypothesis that tACS mechanisms vary according to stimulation duration. This can be addressed in future experiments, where the stimulation duration can be varied as an independent variable to systematically map out its relationship with the boost in entrainment, changes in coherence and other short-term plasticity related changes.

## Conflict of interest statement

The author declares that the research was conducted in the absence of any commercial or financial relationships that could be construed as a potential conflict of interest.
